# A Lesson Learned in Managing Traumatic Cervical Neurofibromatosis Type 1

**DOI:** 10.7759/cureus.80584

**Published:** 2025-03-14

**Authors:** Sharifah Fatimah Syahirah Syed Afandi, Rohaida Ibrahim, Mohd Razif Mohamad Yunus

**Affiliations:** 1 Department of Otolaryngology-Head and Neck Surgery, Faculty of Medicine, Universiti Kebangsaan Malaysia, Kuala Lumpur, MYS; 2 Department of Otolaryngology, Hospital Sultanah Nur Zahirah, Kuala Terengganu, MYS; 3 Department of Otolaryngology-Head and Neck Surgery, Universiti Kebangsaan Malaysia Medical Centre, Kuala Lumpur, MYS

**Keywords:** diagnosis, management, neck, neurofibromatosis type 1, presentation

## Abstract

Neurofibromatosis type 1 (NF-1) is also known as von Recklinghausen disease. It is a rare autosomal dominant disorder that affects the skin and nervous system. The head and neck region is one of the potential areas for the involvement of neurofibromatosis due to the unique anatomical compartmentalization of the central and peripheral nervous systems. Neurofibromas in the head and neck region are primarily found within the soft tissues. NF-1 can be diagnosed during childhood because characteristic cutaneous manifestations are typically present during childhood. In this report, we discuss a case involving a 30-year-old woman diagnosed with NF-1. She presented with right neck swelling preceded by a history of neck trauma. She had a history of numerous café-au-lait spots in the thorax, neck, and facial region. The diagnosis of NF-1 was made based on the radiographic findings and National Institutes of Health (NIH) criteria. The patient was treated conservatively with follow-up.

## Introduction

Neurofibromatoses are a group of genetic disorders that affect nerve tissues. Neurofibromatosis is classified into neurofibromatosis type 1 (NF-1), neurofibromatosis type 2 (NF-2), and schwannomatosis, with NF-1 being the most prevalent and primarily impacting both the central and peripheral nervous systems.

NF-1 was first described in 1882 by Friedrich von Recklinghausen [1]. NF-1, also known as von Recklinghausen disease, is an autosomal dominant condition resulting from a fundamental defect in neural crest cells, leading to the development of ectodermal and mesodermal derivatives that impact approximately one in every 2,500-3,000 births [1].

NF-1 is typically inherited, although 30-50% of cases occur due to a spontaneous mutation in the gene [2]. Tumour suppressor gene defect can result in NF-1, and it will predispose the individual to malignancy [2]. NF-1 affects people equally regardless of race, ethnic groups, and gender [2]. NF-1 can be diagnosed at a young age because the characteristics of cutaneous manifestation typically present during childhood and these signs can be easily identified by clinical inspection [3]. Clinicians should be aware of these signs, and those who discover these cutaneous lesions should refer the patient to a neurologist for laboratory and genetic testing.

Based on the National Institutes of Health (NIH), the clinical criteria for the diagnosis of NF-1 are the following, of which at least two should be present: the presence of six or more café-au-lait spots, two or more neurofibromas of any kind, or a single plexiform neurofibroma, freckling in the axillary or inguinal region, optic glioma, two or more Lisch nodules (iris hamartomas), a distinctive osseous lesion such as sphenoid dysplasia or thinning of long bone cortex with or without pseudoarthrosis, and a first-degree relative with NF-1 [1,4,5].

## Case presentation

**Figure 1:**
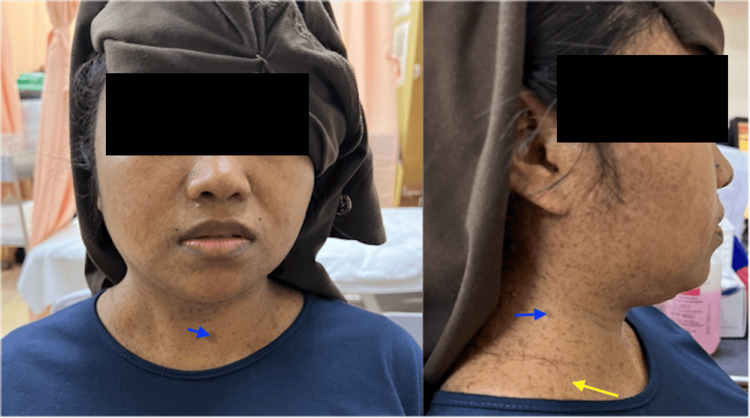
The patient with right neck swelling (yellow arrow) and numerous café-au-lait skin spots (blue arrow) disseminated at the thorax, neck and facial region.

A 30-year-old lady presented with painful right neck swelling associated with dysphagia in the past five days prior to the presentation. She had been punched over the right neck area one week prior to the swelling. Physical examination revealed a right neck swelling at the posterior triangle region, with a size of 5 cm × 3 cm, firm in consistency, and tender on palpation. There were numerous café-au-lait skin spots disseminated at the thorax, neck, and facial region (Figure 1).

**Figure 2:**
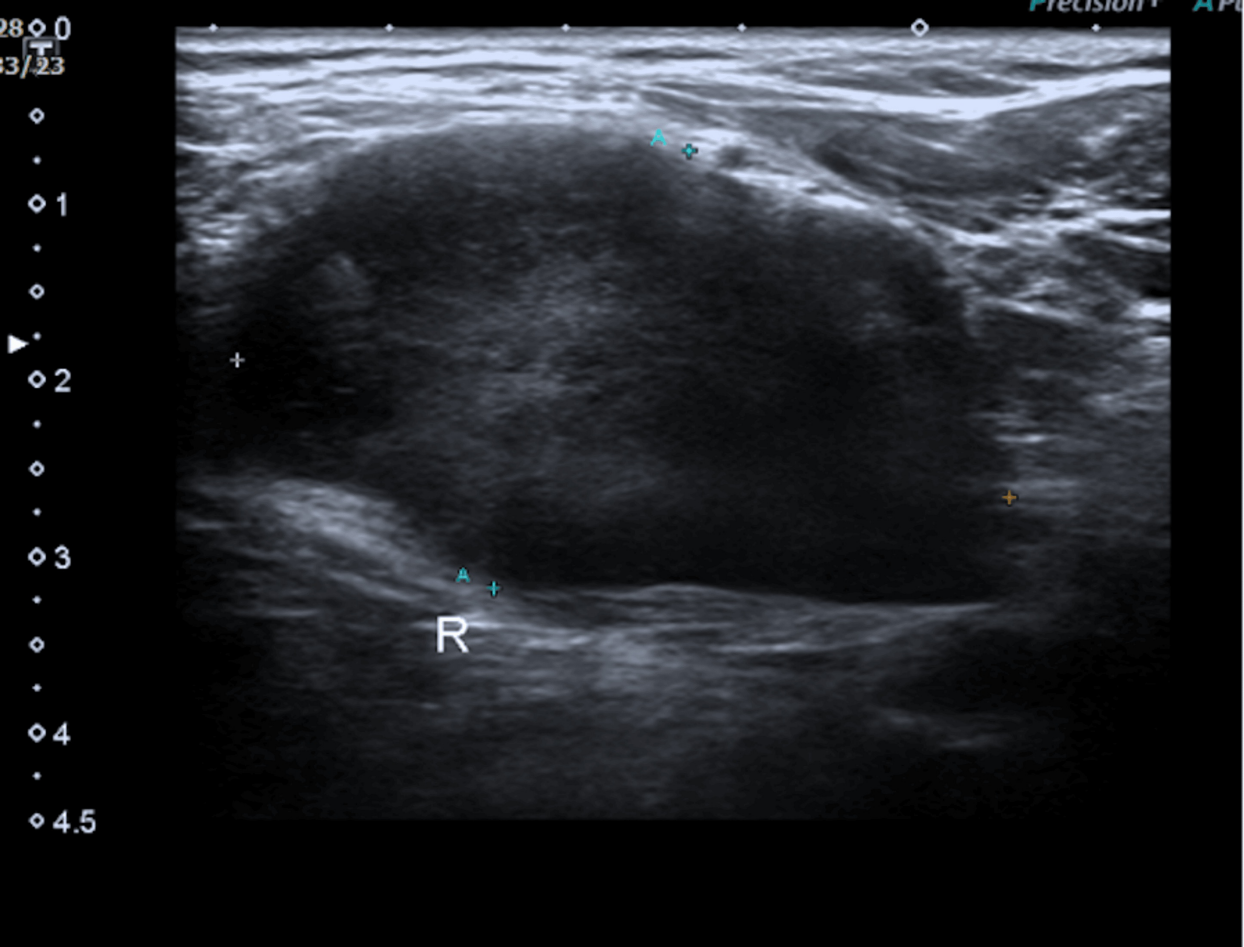
An ultrasound of the neck revealed a large, well-defined, heterogeneous, hypoechoic lesion measuring 2.7 cm × 4.4 cm (AP × W) seen within the right sternocleidomastoid muscle. AP × W: anteroposterior length × width

Ultrasound of the neck was performed and revealed a large, well-defined, heterogeneous, hypoechoic lesion measuring 2.7 cm × 4.4 cm (anteroposterior length (AP) × width (W)) seen within the right sternocleidomastoid muscle with features representing hematoma or benign cystic lesion (Figure 2).

**Figure 3:**
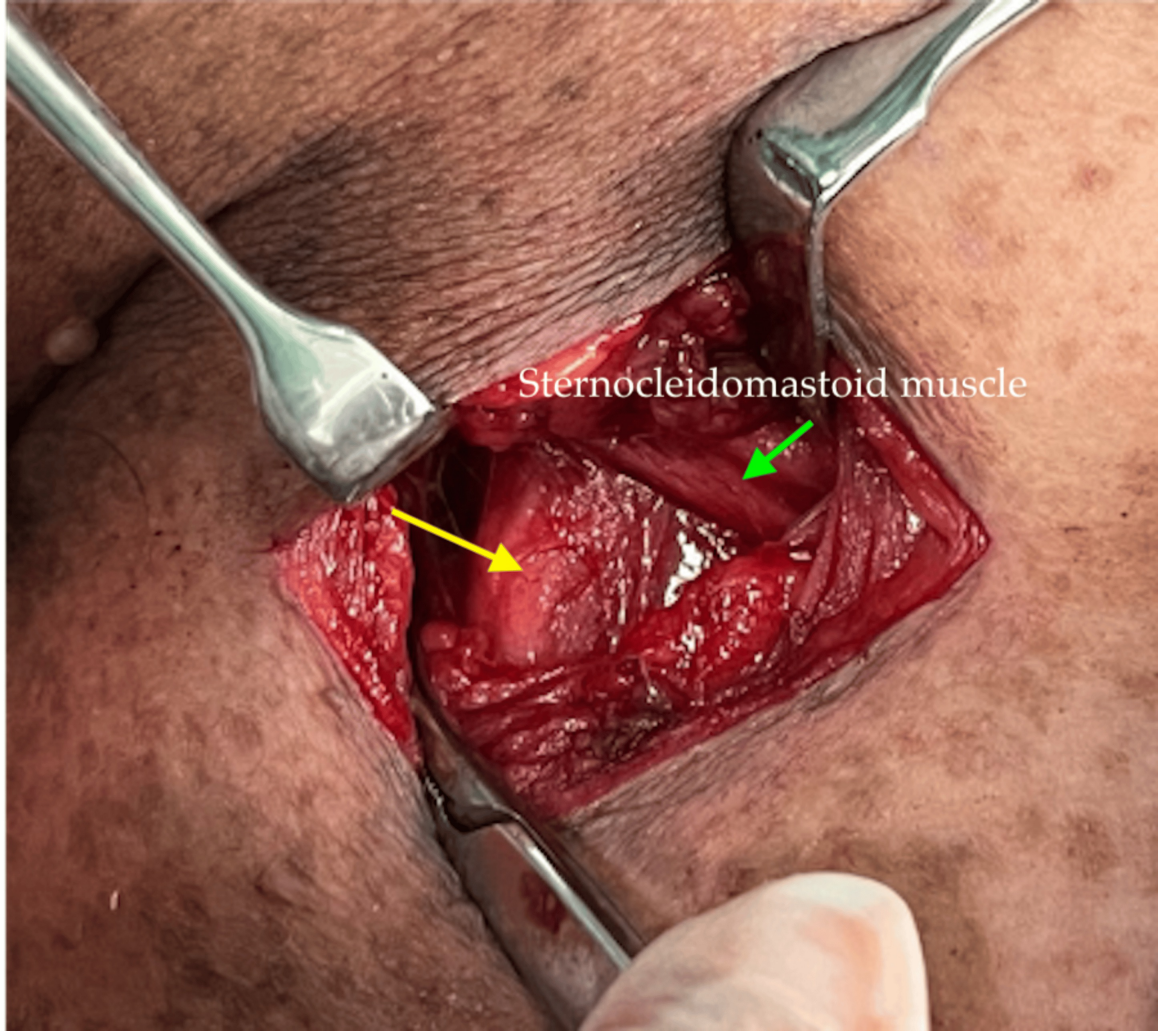
A fusiform-like mass (yellow arrow) located at the posterior border of the lower third of the right sternocleidomastoid muscle (green arrow).

She was initially treated for right neck infected hematoma and was subjected for incision and drainage. However, intraoperative findings revealed a fusiform-like mass located at the posterior border of the lower third of the right sternocleidomastoid muscle (Figure 3). The procedure of incision and drainage was abandoned, and she was subjected for further imaging.

**Figure 4:**
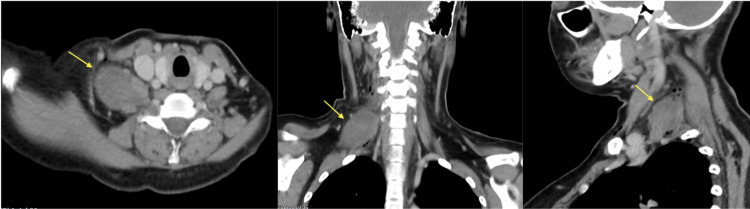
CT scan of the neck with contrast showed a fusiform, heterogeneous enhanced soft tissue lesion (yellow arrow) arising from the right C5 and C6 cervical nerve root causing the relative widening of the neural foramina fullness of the right C5/C6 and C6/C7.

Subsequently, a CT scan of the neck was performed, showing features suggestive of a soft tissue lesion arising from the root C5 and C6 nerves, likely a peripheral nerve sheath tumour (Figure 4). 

**Figure 5:**
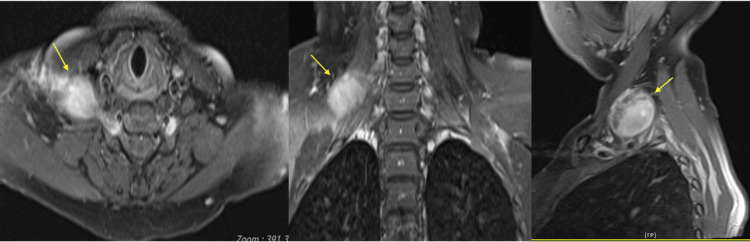
T2-weighted MRI of the neck with contrast demonstrates heterogeneously hyperintense lesion at the right cervical paravertebral region (yellow arrow).

MRI of the brain and neck was done and revealed a well-defined fusiform lesion at the right cervical paravertebral region from C5 to C8 levels, located between the right anterior scalene and middle scalene muscle, measuring 3 cm × 3.9 cm × 3.5 cm (AP × W × craniocaudal length (CC)) with an extension of mass through the right C5/C6 neural foramen causing the widening of the C5 and C6 neural foramina. The T2-weighted image (T2WI) demonstrates a heterogeneously hyperintense lesion with heterogeneous enhancement post-contrast (Figure 5).

A diagnosis of NF-1 was made. The patient was offered for surgical excision of the tumour; however, she opted for conservative management in view of improving symptoms.

## Discussion

The variability of presentation and the rarity of NF-1 lead to the misleading diagnosis and thus result in inappropriate management.

The classical lesion of NF-1 is neurofibroma. Neurofibromas are solid masses arising from the neuronal tissue, and they can be either simple or plexiform. Plexiform neurofibromas are found in the regions of the head, neck, face, and larynx in approximately 50% of cases of NF-1 [2,6]. The clinical manifestation of NF-1 is widely variable, as an individual with NF-1 can present with reduced fine motor skills and motor speed, pain or weakness in the upper and lower extremities, dysphagia, compromised airway, enlarged soft tissue mass, paralysis, spastic hemiparesis, and spinal deformities [2]. Radiculopathy and motor loss are the consequences of spinal nerve root compression by neurofibromas. Individuals with neurofibromas compressing on the head and cervical structures can also present with headaches, facial paralysis, decreased cervical mobility, muscle imbalances, and postural asymmetries.

Spinal manifestations are a common presentation and occur in 60% of NF-1 patients, and another 40% of adults with NF-1 have internal neurofibromas, which cannot be seen on physical examination and are likely to be asymptomatic [3]. Patients may experience neck and back pain as well as clinical neurological deficit because of the spinal deformity due to the presence of neurofibromas [3]. 

Cutaneous abnormalities are the common signs, and the café-au-lait spots are the first clinical evidence of the disease. A high index of suspicion should be raised by clinicians seeing these cutaneous lesions, and further investigation by laboratory and genetic testing should be performed. 

Neurofibroma can undergo malignant transformation. Malignant peripheral nerve sheath tumours are rare, causing 5-10% of all adult soft tissue sarcomas to be related to NF-1, with 25-50% of those cases linked to the condition [5]. The occurrence rate is 8-13% among NF-1 patients [4]. Patients with NF-1 at a younger age and larger tumours tend to undergo malignant transformation [4]. 

Radiographs are of limited value in evaluating malignant peripheral nerve sheath tumours. Ultrasonography has limited usefulness for assessing deeply located lesions, while CT is effective for defining the tumour's extent, identifying bone involvement, and assisting in preoperative planning [5]. Fluorodeoxyglucose-positron emission tomography (FDG-PET) plays a vital role in distinguishing between benign and malignant neurofibromas and is also useful for staging and directing biopsies towards the tumour with the highest grade [5]. On the other hand, MRI is regarded as the preferred imaging technique for assessing malignant peripheral nerve sheath tumours due to its superior soft tissue resolution and capacity to characterise different tumour components while accurately defining surgical margins [5]. Imaging characteristics indicative of malignant transformation include an enlarging mass, peripheral enhancement, perilesional oedema, and increased heterogeneity within the tumour [5].

The significant variability in clinical presentation, unpredictable progression of NF-1, and uncertain growth rates of plexiform neurofibromas contributed to an unclear overall medical prognosis for this patient [2]. Approximately one-third of individuals with NF-1 will face severe complications, whereas around 50% will experience only mild issues [2]. 

Surgical removal or resection of the tumour is performed in severe cases of NF-1, when the tumour is malignant, compressing the vital structures, causing recurrent symptoms, or aesthetically unappealing [2]. In cases where the tumours do not meet the criteria for surgery, conservative treatment may be effective in alleviating or resolving related secondary conditions and symptoms [2]. Radiotherapy and radiosurgery are likely to locally control neurofibromas in patients who require treatment but are not good candidates for complete resection [7]. Radiotherapy should be considered in neurofibromatosis with the progression of central nervous system tumours with the evidence of imaging [8].

This case study describes the head and neck presentation of NF-1 and the lesson learned by the clinicians in establishing the diagnosis. In this case study, the patient presented with a painful right neck swelling preceded by a history of neck trauma. From the history, physical examination, and ultrasound findings, the diagnosis of right neck hematoma was made, and the patient was subjected for incision and drainage. However, intraoperative findings revealed a fusiform-like mass located at the posterior border of the lower third of the right sternocleidomastoid muscle. The procedure was abandoned, and a CT scan of the neck was performed to identify the origin of the tumour and to delineate the extent of the tumour. The average size of a malignant peripheral nerve sheath tumour is usually above 5 cm, probably due to its rapid growth.

A CT scan of the neck revealed a fusiform, heterogeneous enhanced soft tissue lesion arising from the right C5 and C6 cervical nerve roots, causing the relative widening of the neural foramina fullness of the right C5/C6 and C6/C7. The lesion measured 2.9 cm × 4.6 cm × 5.3 cm. No calcification or fat component is seen within this lesion. A small enhancing cutaneous lesion over the right supra-clavicular region is suggestive of cutaneous neurofibroma. Brain and cervical MRI was performed to look for tumour extension and to evaluate for malignant transformation. 

Based on the patient's presentation, we can conclude that the painful neck swelling is exaggerated by the neck trauma. The painful neck is a cervical consequence of NF-1, which is due to the soft tissue lesion. Neurofibromas that lead to spinal deformities are linked to cervical pain and limited movement in the cervical region [2].

The patient was treated conservatively as her symptoms were improving, and she did not experience repeated symptoms. Physical therapy management may be beneficial, and collaboration with other healthcare providers and physical therapists is crucial for the conservative management of individuals with NF-1 [2]. Through physiotherapy and effective pain management, patients can experience improvements in functional limitations and restrictions in their home, work, or recreational activities [2]. Thus, the patient can carry on with her daily activities comfortably and subsequently be able to return to her previous activities.

## Conclusions

NF-1 is a rare autosomal dominant disorder which affects the skin and nervous system. The head and neck region is one of the potential areas for the involvement of neurofibromatosis. Thus, retrospectively, the lesson to learn from this case is that, in managing traumatic injury of NF-1, we should perform a CT scan as the presence of café-au-lait spots with possible bleeding from the intracystic lesion can lead to pain. Physicians should raise the suspicion of NF-1 as one of the differential diagnoses in a patient presenting with neck swelling and meeting the criteria of diagnosis based on the NIH, supported by the evidence from the radiographic findings. Surgical removal can be done in selected patients, and conservative management may be beneficial in a patient where the tumours do not meet the criteria for surgery. A multidisciplinary management is required in managing NF-1. Good physical therapy and pain management will improve the quality of life, and the patient will be able to return to her previous activities.

## References

[1] Nallanchakrava S, Mallela MK, Jeenepalli VS, Niharika HM (2020). A rare case report of neurofibromatosis type 1 in a 12-year-old child: a 15-month follow-up. J Oral Maxillofac Pathol.

[2] Helmers KM, Irwin KE (2009). Physical therapy as conservative management for cervical pain and headaches in an adolescent with neurofibromatosis type 1: a case study. J Neurol Phys Ther.

[3] Mignelli J, Tollefson LJ, Stefanowicz E (2021). Conservative management of neck and thoracic pain in an adult with neurofibromatosis-1. J Can Chiropr Assoc.

[4] Hillier JC, Moskovic E (2005). The soft-tissue manifestations of neurofibromatosis type 1. Clin Radiol.

[5] Chew DC, Zhao DB, Sittampalam K, Kumar SK (2020). Malignant transformation in a sciatic plexiform neurofibroma in neurofibromatosis type 1 - imaging features that aid diagnosis. J Radiol Case Rep.

[6] Hersh JH (2008). Health supervision for children with neurofibromatosis. Pediatrics.

[7] Yu YH, Wu JT, Ye J, Chen MX (2016). Radiological findings of malignant peripheral nerve sheath tumor: reports of six cases and review of literature. World J Surg Oncol.

[8] Chopra R, Morris CG, Friedman WA, Mendenhall WM (2005). Radiotherapy and radiosurgery for benign neurofibromas. Am J Clin Oncol.

